# A review: Circadian Rhythm Dysfunction and Bipolar Disorder

**DOI:** 10.1192/j.eurpsy.2022.1035

**Published:** 2022-09-01

**Authors:** M. Ribeiro, A. Lourenço, M. Lemos, J. Bastos, J.M. Pereira

**Affiliations:** 1 Centro Hospitalar Lisboa Norte, Psychiatry, Lisboa, Portugal; 2 Hospital Prof. Doutor Fernando Fonseca, Departamento De Saúde Mental, Amadora, Portugal

**Keywords:** circadian rhythm, bipolar disorder, sleep

## Abstract

**Introduction:**

Circadian rhythm (CR) dysfunction is a prominent feature in bipolar disorder (BD) and sleep disturbances are characteristic, although not essential to the diagnosis.

**Objectives:**

To review the literature regarding the CR dysfunction and its impact on the onset and clinical course of BD.

**Methods:**

We conducted a MEDLINE search using bipolar disorder, circadian rhythm and sleep as keywords, selecting studies written in English.

**Results:**

CR dysfunction is a trait marker of BD. It’s known that during depressive episodes insomnia is present, with difficulty falling asleep/ maintaining sleep and early awakening. Regarding mania, decreased need for sleep is a critical marker. During the euthymic period significant alterations in sleep pattern have been described. It’s also known that changes in the sleep pattern occur prior to those in mood patterns, indicating that sleep dysregulation may trigger the onset of mood episodes or relapses. Therefore, CR disruption may be associated with the pathophysiology of BD and some factors have already been identified: irregularity of the sleep-wake rhythm, eveningness chronotype, abnormality of melatonin secretion, vulnerability of clock genes and the irregularity of social *zeitgeber.*

**Conclusions:**

Disturbances of sleep are pervasive, and an essential feature of BD, worse during mood episodes, but still present during euthymic periods. It remains to determine whether circadian rhythm dysfunction is a trait marker or mood state dependent. Further studies are warranted to clarify this association.

**Disclosure:**

No significant relationships.

